# Deep learning enables the differentiation between early and late stages of hip avascular necrosis

**DOI:** 10.1007/s00330-023-10104-5

**Published:** 2023-08-15

**Authors:** Michail E. Klontzas, Evangelia E. Vassalou, Konstantinos Spanakis, Felix Meurer, Klaus Woertler, Aristeidis Zibis, Kostas Marias, Apostolos H. Karantanas

**Affiliations:** 1 Department of Radiology, School of Medicine, University of Crete, Voutes Campus, Heraklion, Greece; 2 Department of Medical Imaging, University Hospital of Heraklion, 71110 Voutes, Crete Greece; 3 Computational BioMedicine Laboratory, Institute of Computer Science, Foundation for Research and Technology (FORTH), Heraklion, Crete Greece; 4 Advanced Hybrid Imaging Systems, Institute of Computer Science, Foundation for Research and Technology (FORTH), Nikolaou Plastira 100, 70013 Heraklion, Crete Greece; 5 Musculoskeletal Radiology Section, TUM School of Medicine, Technical University of Munich, Ismaninger Str 22, 81675 Munich, Germany; 6 Department of Anatomy, Medical School, University of Thessaly, Neofytou 9 St., 41223 Larissa, Greece; 7 Department of Electrical & Computer Engineering, Hellenic Mediterranean University, Heraklion, Crete Greece

**Keywords:** Deep learning, Artificial intelligence, Hip, Necrosis, avascular of bone, Osteoarthritis

## Abstract

**Objectives:**

To develop a deep learning methodology that distinguishes early from late stages of avascular necrosis of the hip (AVN) to determine treatment decisions.

**Methods:**

Three convolutional neural networks (CNNs) VGG-16, Inception ResnetV2, InceptionV3 were trained with transfer learning (ImageNet) and finetuned with a retrospectively collected cohort of (*n *= 104) MRI examinations of AVN patients, to differentiate between early (ARCO 1–2) and late (ARCO 3–4) stages. A consensus CNN ensemble decision was recorded as the agreement of at least two CNNs. CNN and ensemble performance was benchmarked on an independent cohort of 49 patients from another country and was compared to the performance of two MSK radiologists. CNN performance was expressed with areas under the curve (AUC), the respective 95% confidence intervals (CIs) and precision, and recall and f1-scores. AUCs were compared with DeLong’s test.

**Results:**

On internal testing, Inception-ResnetV2 achieved the highest individual performance with an AUC of 99.7% (95%CI 99–100%), followed by InceptionV3 and VGG-16 with AUCs of 99.3% (95%CI 98.4–100%) and 97.3% (95%CI 95.5–99.2%) respectively. The CNN ensemble the same AUCs Inception ResnetV2. On external validation, model performance dropped with VGG-16 achieving the highest individual AUC of 78.9% (95%CI 51.6–79.6%) The best external performance was achieved by the model ensemble with an AUC of 85.5% (95%CI 72.2–93.9%). No significant difference was found between the CNN ensemble and expert MSK radiologists (*p *= 0.22 and 0.092 respectively).

**Conclusion:**

An externally validated CNN ensemble accurately distinguishes between the early and late stages of AVN and has comparable performance to expert MSK radiologists.

**Clinical relevance statement:**

This paper introduces the use of deep learning for the differentiation between early and late avascular necrosis of the hip, assisting in a complex clinical decision that can determine the choice between conservative and surgical treatment.

**Key Points:**

*• A convolutional neural network ensemble achieved excellent performance in distinguishing between early and late avascular necrosis.*

*• The performance of the deep learning method was similar to the performance of expert readers.*

**Supplementary information:**

The online version contains supplementary material available at 10.1007/s00330-023-10104-5.

## Introduction

Avascular necrosis (AVN) of the hip affects approximately 20,000 patients every year in the USA and is the most common cause of total hip arthroplasty (THA) at young ages, demonstrated with bilateral involvement in > 70% of patients [1]. In cases where AVN is left untreated, it progresses to joint collapse and secondary osteoarthritis with THA being the only treatment option. However, in the early stages of the disease prior to articular collapse, joint preservation techniques (core decompression, vascularized grafting, etc) are available with the potential to avoid THA [2]. Therefore, differentiation between early and late AVN is of utmost importance for appropriate treatment selection.

Several AVN staging systems exist with the system of the Association Research Circulation Osseous (ARCO) [3] being the most commonly used. ARCO staging is recommended in the latest (2019) international guidelines on the management of AVN [4]. The latest version of ARCO defines four main stages, with joint preservation techniques being available for the two first stages (ARCO < 3) whereas hip replacement being the recommended treatment for terminal disease (ARCO 3–4). Nonetheless, distinguishing between ARCO 2 (early) and ARCO 3A (late) is an extremely challenging task, requiring significant expertise in musculoskeletal radiology and a combination of imaging findings including indications of loss of femoral head sphericity and the presence of a subchondral fracture [4–6].

Artificial intelligence has been previously used for the diagnosis of AVN on plain radiographs [7] and MRI [8], to identify factors increasing the risk for collapse [9], and to differentiate late AVN from other causes of proximal femoral bone marrow edema such as transient osteoporosis [10, 11]. Attempts have been also recently made to quantify the necrotic volume and surface area in an attempt to associate this with the stage of AVN [12]. However, quantification of the necrotic part volume is not part of any clinically relevant classification system and volume cut-offs have not been set to levels that will define optimal treatment.

The aim of this study was to develop a deep learning methodology to differentiate between the early (ARCO 1 and 2) and late (ARCO 3 and 4) stages of AVN. For this purpose, convolutional neural networks (CNNs) have been trained with a transfer learning methodology and finetuned with the use of a cohort of patients with AVN. The algorithm was internally tested and then subjected to external validation on an international cohort of AVN patients and its performance was benchmarked against the performance of musculoskeletal radiologists. The development of such a model would be invaluable in assisting clinical decisions between joint preservation surgery and total hip arthroplasty.

## Materials and methods

### Patients

A multi-institutional dataset (UHH cohort) of 104 consecutive hips (67 patients) with AVN was retrospectively compiled. The cohort contained 36 cases of early and 68 late cases of AVN. A combination of transfer learning and data augmentation was used to address the small size of the dataset as proposed by Candemir et al [13] (described below). All patients were evaluated in our specialized musculoskeletal imaging clinic and the second opinion bone marrow imaging clinic receiving domestic and international referrals in complicated hip cases. Cases were prospectively collected in an AVN registry and then imaging data were retrospectively retrieved based on this registry of our MSK clinics. This cohort has been previously used to develop deep learning and radiomics methodology for the differentiation between avascular necrosis and transient osteoporosis of the hip [10, 11]. Exclusion criteria included hips with tumours, prior trauma, infection, inflammatory arthropathies, or surgery at the hip of interest as well as insufficient image quality. Hips with extensive red marrow infiltration have been also excluded to avoid confounding effects imitating bone marrow edema of the proximal hip. This cohort was used for the training and internal testing of CNNs.

**Fig. 1:**
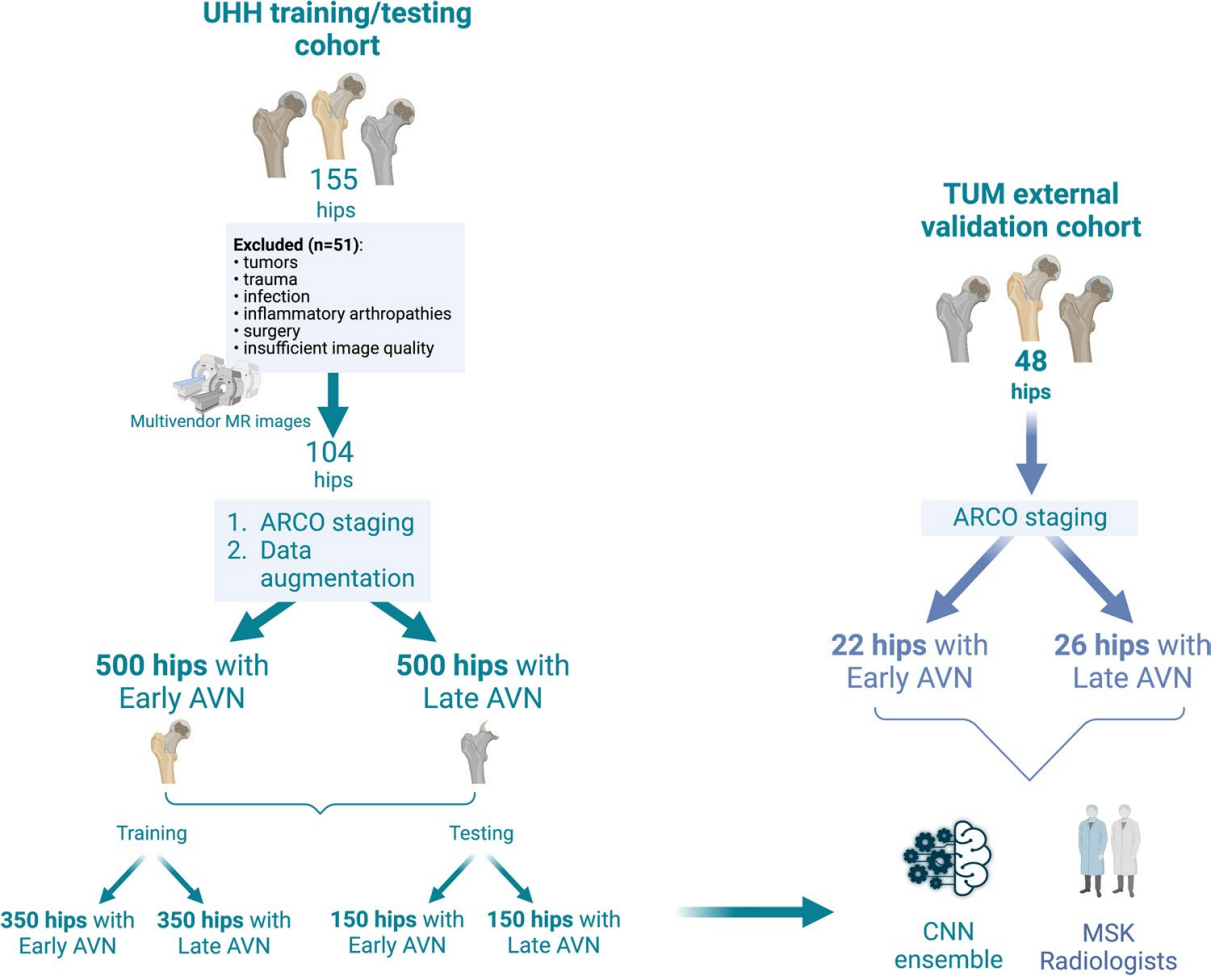
Flow diagram explaining the characteristics of the UHH and TUM cohorts for training/testing and external validation respectively. AVN: avascular necrosis of the hip; CNN: convolutional neural networks; MSK: musculoskeletal; ARCO: Association Research Circulation Osseous (created with biorender.com)

External validation of the developed deep learning methodology was performed using an independent anonymized cohort from a center located in another country (TUM cohort, *n *= 49 hips) which was retrospectively selected based on the same criteria (Fig. 1). The study has been performed according to the Helsinki Declaration and has been approved by the ethical committee of the University Hospital of Heraklion (Ref. No. 360/08/29-04-2020). Informed consent has been waived for the retrospective use of anonymized data. This manuscript was prepared according to the Checklist for Artificial Intelligence in Medical Imaging (CLAIM) [14] and the Standards for Reporting of Diagnostic accuracy studies (STARD) checklist [15].

### MR imaging and ground truth ARCO staging

**Fig. 2:**
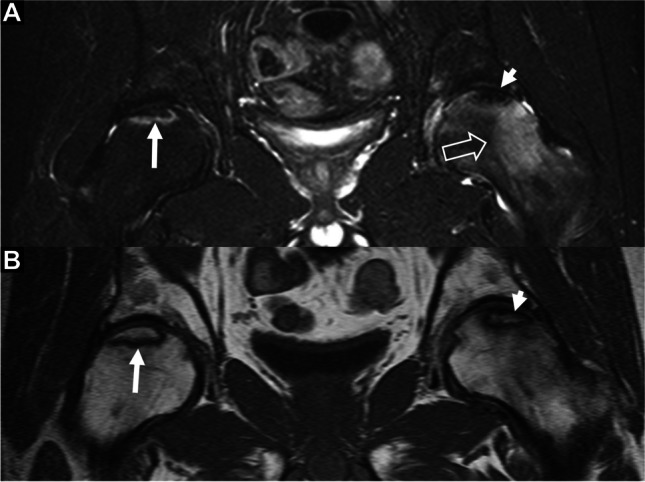
Coronal STIR (**A**) and T1-w (**B**) MR images, showing bilateral idiopathic avascular necrosis, in a 43-year-old male presenting with a left painful hip. The lesion on the right hip (arrows), is asymptomatic and is occult on plain radiographs (not shown). The lesion on the left is associated with bone marrow edema (open arrow) secondary to mild articular surface flattening (short arrows). According to ARCO classification, the right lesion is stage I and the left stage III

Ground truth staging of AVN was established based on imaging, according to the ARCO classification [3]. This is currently the gold standard practice for clinical diagnosis and staging since the diagnosis of AVN does not warrant biopsy. Ground truth staging was performed independently by two MSK radiologists (40 and 10 years of experience, respectively) and in cases of disagreement, final stage was defined by consensus. To ensure accurate ground truth grading the experts had access to the whole MRI protocol including T1-w, STIR or PD/T2 fs, and high-resolution 3D gradient echo images. AVN was diagnosed by the presence of the “band-like” sign on T1-w images [16]. Subsequently, two groups of hips were defined based on T1-w, STIR, and high-resolution 3D gradient echo images: (i) cases with a subchondral fracture, cases with loss of head sphericity and/or associated bone marrow edema, and cases with signs of secondary osteoarthritis were classified as “late AVN” (ARCO 3–4) and (ii) cases without the aforementioned findings were classified as “early AVN” (ARCO 1-2) (Fig. 2) [1, 17, 18].

### Data pre-processing and augmentation

Mid-coronal STIR images through each femoral head were used for model training, testing, and validation. Images were resized to 150 × 150 pixels and then images were randomly split 70:30 in training: testing sets. Data harmonization and bias correction were performed by matching image histograms to account for intra-scanner variability and achieve gray-level normalization. In order to eliminate group imbalance bias and to expose the model to additional training/testing data, images were augmented using rotation of 10° (clockwise and anti-clockwise) as well as horizontal image flipping. The final training and testing datasets consisted of a total of 350 training and 150 testing images for each of the two groups (early vs late AVN) (Fig. 1).

### Convolutional neural network development and external validation

A CNN ensemble was used as previously described [19]. Briefly, transfer learning was applied by obtaining the initial weights of three individual CNN architectures, VGG-16, InceptionV3, and Inception-ResNetV2, training first with the ImageNet dataset followed by weight freezing and final trainable layer finetuning with the use of our training dataset [20]. Network performance was subsequently evaluated with the use of the UHH testing dataset. A consensus ensemble decision of the three CNNs was recorded as the agreement of at least two out of three CNNs. To further benchmark the performance of the CNN ensemble, the resulting model was externally validated on a set of 49 hips from a radiology department of another country (TUM dataset). Images were resized and used without any further pre-processing. Ground truth for the TUM dataset was established with the same method as for the UHH dataset External validation images were also assessed by two experienced MSK radiologists (10 and 7 years of MSK experience) blinded to the results of the ensemble and their performance was compared to the performance of the ensemble. CNNs were trained for 100 epochs with early stopping at 10 rounds to avoid overfitting. Deep learning was performed with Python v.3.8, the Keras framework, and the TensorFlow backend on a Windows 10 Pro workstation with 32 GB RAM, Intel i7-10700F @2,9 GHz CPU, and NVIDIA GeForce RTX 2060 Super 8GB GPU.

### Statistical analysis

CNN performance was evaluated using precision, recall, and f1-scores for each individual CNN and the ensemble. CNN and MSK expert performance was also assessed with receiver operating characteristics (ROC) curves and the respective area under the curve (AUC) with 95% confidence intervals for the AUC calculated with bootstrapping with the use of the pROC package [21] as implemented in R (v.4.03, https://www.R-project.org/). A single threshold value at 0.5 was used for the ROC curves given the fact that upon augmentation groups were balanced. Comparisons between the AUCs of the models and experts were performed with DeLong’s test [22].

## Results

### Individual and ensemble CNN performance

**Fig. 3:**
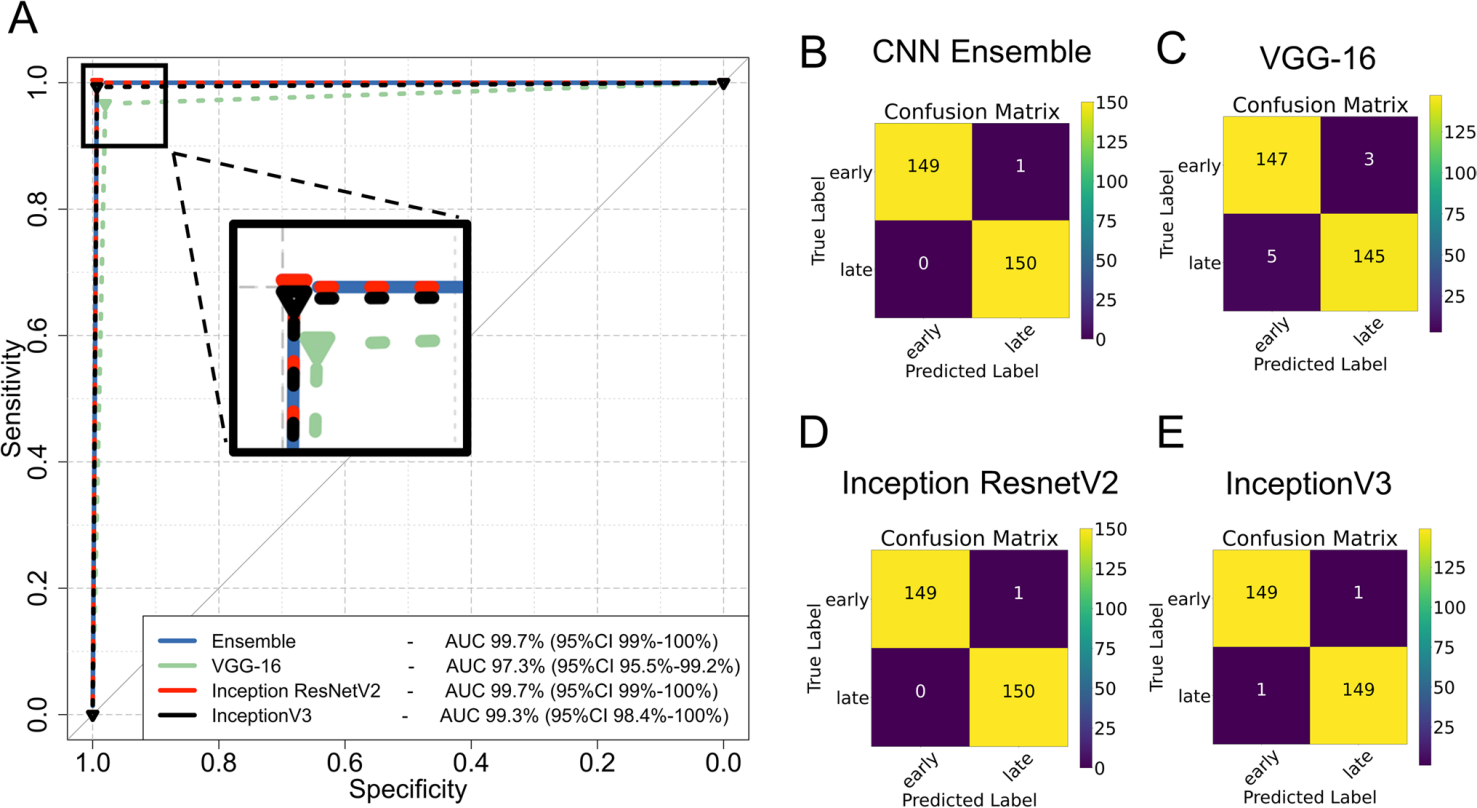
Performance of individual CNNs and their ensemble on the UHH cohort. Model performance is demonstrated on Receiver Operating Characteristics (ROC) curves of the ensemble and the CNNs (**A**) and confusion matrices for the CNN ensemble (**B**) and individual CNNs (**C**–**E**). Insert on (**A**) magnifies the upper left corner of the ROC graph. AUC: area under the curve

The average age of patients in the training/testing (UHH) cohort was 43.7 ± 14.7 years including 48 female and 56 male patients with 56 right and 48 left hips. Each CNN architecture was initially subjected to internal testing with the UHH cohort where Inception-ResnetV2 achieved the highest individual performance with an AUC of 99.7% (95%CI 99–100%), followed by InceptionV3 and VGG-16 with AUCs of 99.3% (95%CI 98.4–100%) and 97.3% (95%CI 95.5–99.2%) respectively. VGG-16 had the highest number of misclassified cases with three early cases misclassified as late and five late cases misclassified as early. The model ensemble achieved an AUC similar to Inception ResnetV2 with only one early case misclassified as late (Fig. 3 and Table 1). Training and validation accuracy/loss plots were observed to ensure that overfitting was avoided (Supplementary Fig. 1).

**Table 1:** Performance metrics of individual models and the neural network ensemble

		Internal Testing (UHH cohort)				External Validation (TUM cohort)			
	Group	AUC	Precision	Recall	f1-score	AUC	Precision	Recall	f1-score
Model ensemble	Early AVN	99.7% (99–100%)	1	0.99	1	85.5% (72.2–93.9%)	0.86	0.82	0.84
	Late AVN		0.99	1	1		0.85	0.88	0.87
VGG-16	Early AVN	97.3% (95.5–99.2%)	0.97	0.98	0.97	78.9% (51.6–79.6%)	0.58	1	0.73
	Late AVN		0.98	0.97	0.97		1	0.38	0.56
InceptionV3	Early AVN	99.3% (98.4–100%)	0.99	0.99	0.99	74.8% (58.1–84.7%)	0.8	0.55	0.65
	Late AVN		0.99	0.99	0.99		0.7	0.88	0.78
Inception ResNetV2	Early AVN	99.7% (99–100%)	1	0.99	1	76.59% (58.1–84.7%)	0.85	0.5	0.63
	Late AVN		0.99	1	1		0.69	0.92	0.79

*AUC* area under the curve; *AVN a*vascular necrosis of the hip; *AUC* is presented as a percentage with a range of 95% confidence interval

**Fig. 4:**
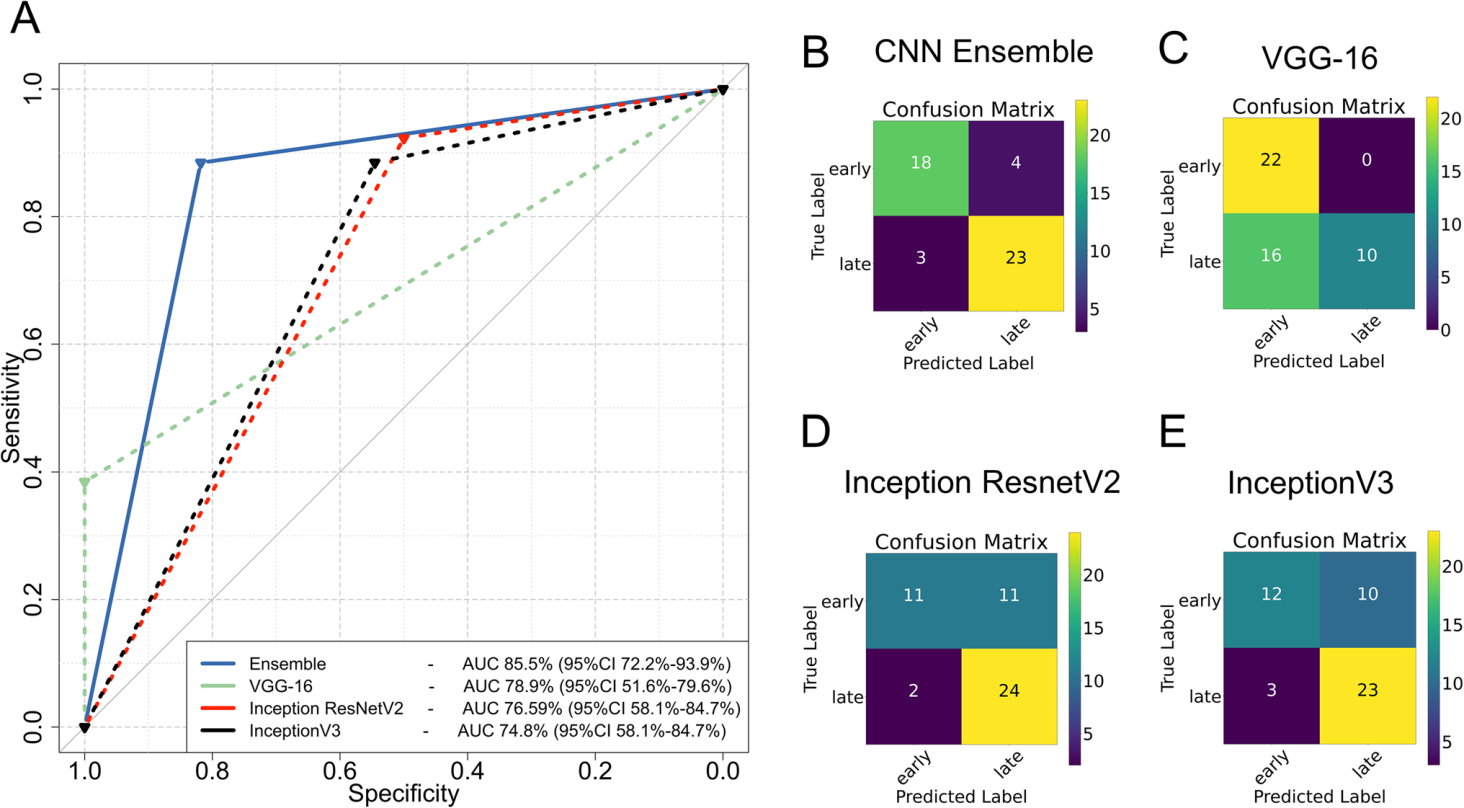
Performance of individual CNNs and their ensemble on the external validation TUM cohort. Model performance is demonstrated on Receiver Operating Characteristics (ROC) curves of the ensemble and the CNNs (**A**) and confusion matrices for the CNN ensemble (**B**) and individual CNNs (**C**–**E**). AUC: area under the curve

The performance of CNNs dropped when benchmarked with the TUM external validation cohort. VGG-16 achieved the highest individual AUC of 78.9% (95%CI 51.6–79.6%) followed by InceptionV3 and Inception ResnetV2 with AUCs of 74.8% (95%CI 58.1–84.7%) and 76.59% (95%CI 58.1–84.7%) respectively. Despite the performance drop, VGG-16 exhibited excellent precision for the diagnosis of late AVN and recall for the diagnosis of early AVN without any early cases misclassified as late. The best performance was achieved by the model ensemble which achieved an excellent AUC of 85.5% (95%CI 72.2–93.9%) with only 3 late cases misclassified as early and 4 early cases misclassified as late. The performance of the CNN ensemble was significantly higher than all individual CNNs (*p* value 0.014, 0.01, and 0.028 for the comparison of the ensemble to VGG-16, Inception ResnetV2, and InceptionV3 respectively) (Fig. 4 and Table 1).

### Comparison between the CNN ensemble and human readers

**Fig. 5:**
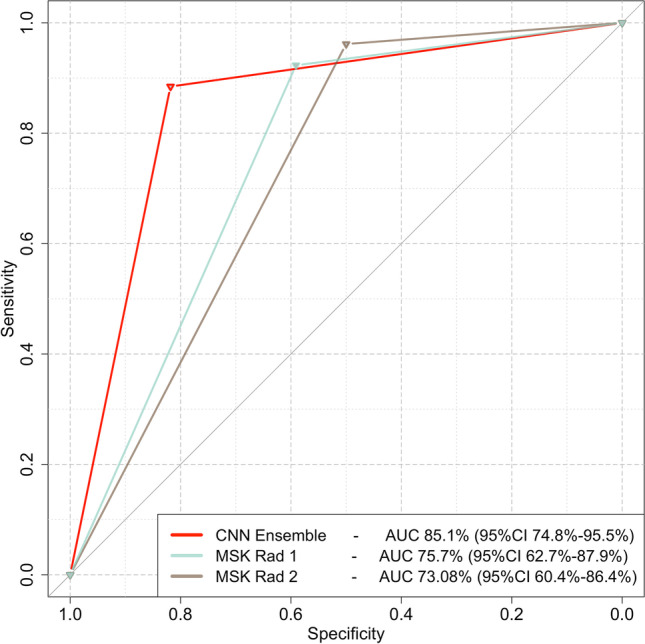
Receiver operating characteristics (ROC) curve comparing the performance of the CNN ensemble and the two expert MSK radiologists on the external validation cohort. AUC: area under the curve; MSK Rad: musculoskeletal radiologist

The performance of the CNN ensemble was compared to the performance of expert readers on the TUM external validation cohort. The first MSK radiologist achieved an AUC of 75.7% (95%CI 62.7–87.9%), whereas the second achieved an AUC of 73.08% (95%CI 60.4–86.4%). No significant difference was found between the performance of each MSK radiologist and the CNN ensemble (*p* value 0.22 and 0.092 for the comparison of the CNN ensemble to the first and second MSK radiologist respectively). Both MSK radiologists achieved excellent recall values for the detection of late AVN (96.1% and 92.3% respectively) with only 1 and 2 late cases misclassified as early for the first and second MSK radiologists respectively (Fig. 5).

## Discussion

Herein, a CNN ensemble was presented that achieved excellent performance in differentiating between early (ARCO 1–2) and late (ARCO 3–4) AVN of the hip. Three individual CNN architectures were trained and a consensus ensemble decision was derived. The excellent performance of the CNN ensemble was confirmed by external validation and was found equal to the performance of experienced MSK radiologists.

The development of a deep learning methodology to differentiate early from late AVN can be of great value in everyday clinical practice. The difficulty of distinguishing between ARCO 2 and 3A has been highlighted in several publications [5, 6, 17] and several indirect findings have been proposed as indicators of late AVN including the presence of bone marrow edema [17], joint effusion, cystic changes, bone resorption [5], and a combination of T2 signal heterogeneity, articular surface irregularity and a necrotic-viable interface with a width >3 mm [6]. Nonetheless, this remains a challenging task, especially for non-experienced radiologists or in cases where high-resolution sequences are not available that have been shown to be suitable for the accurate evaluation of all associated findings of AVN [23]. Given the fact that AVN can be asymptomatic in the early stages [24], it can be randomly identified in pelvic MRI examinations that are not tailored to the evaluation of the proximal femur. Our CNN ensemble achieved excellent performance in distinguishing between early and late AVN only with the use of coronal STIR images. This presents a great advantage in the hands of inexperienced readers and in cases where high-resolution images through the femoral head are not available to allow comprehensive ARCO staging.

Interestingly enough, all individual CNNs presented an important drop in performance when validated on the external TUM cohort. Such a performance drop has been found in the majority of externally validated deep learning studies [25, 26]. External validation is of utmost importance in establishing the “real-world” performance of deep learning algorithms but, alas, it can be found in only 6% of AI manuscripts [27]. Despite the fact that the exact reasons responsible for performance drop during external validation are still largely unknown [25], the size of the training dataset or the number of participating institutions in the training dataset has been shown to have no effect on external performance [25]. Nonetheless, being able to achieve an ensemble AUC > 85% in a dataset acquired in another country provides strong evidence for the generalizability of our method.

MSK radiologists achieved AUCs in the range of 70–75% which reflects the difficulty in staging the disease, especially in the absence of high-resolution images focused on the femoral head which would allow visualization of subchondral fractures equally or better than CT [18]. MSK radiologists were presented with the same coronal STIR images as the ones used for the external validation of our deep learning method. Both MSK experts achieved a high recall (sensitivity) in detecting late AVN whereas the CNN ensemble achieved high precision and recall for both late and early disease. Achieving a similar performance to MSK experts highlights the clinical value of the proposed algorithm especially in the setting of general radiology practices where highly experienced MSK radiologists are not available and protocols are not focused on the evaluation of the hip.

Our work has certain strengths and limitations. The use of a multi-institutional training dataset, the validation on an external dataset, and the comparable performance to expert readers are important advantages of the proposed deep learning methodology. Limitations of the proposed work include the retrospective nature of the study and the limited training dataset. However, we have used transfer learning and data augmentation, which represent strategies suitable for deep learning with small datasets [13], alleviating this limitation as shown by the excellent performance in the internal and external cohorts. Training of the algorithms solely on coronal STIR images could be also considered as a limitation of our study. However, coronal fluid-sensitive sequences are part of most pelvic MRI protocols even when they are not focused on the hips. Such sequences can depict all the features required for ARCO staging including subchondral fractures, bone marrow edema, joint effusion, synovitis, and loss of head sphericity [1]. Therefore, being able to stage the disease based on a sequence present in most settings (even when AVN is an incidental finding), increases the clinical value of our method.

In conclusion, a CNN ensemble has been trained and validated that accurately distinguishes between the early and late stages of AVN. The ensemble performs well in external data from another country and has comparable performance to expert MSK radiologists. This deep learning methodology has the potential to assist the accurate staging of AVN without the need for expertise in MSK radiology ultimately leading to the correct treatment strategy.

### Supplementary Information

Below is the link to the electronic supplementary material.

Supplementary file1 (PDF 167 KB)

## Abbreviations

ARCO: Association Research Circulation Osseous
AVN: Avascular necrosis
CI: Confidence interval
CLAIM: Checklist for Artificial Intelligence in Medical Imaging
CNN: Convolutional neural network
MSK: Musculoskeletal
STARD: Standards for Reporting of Diagnostic accuracy studies

## Declarations

### Guarantor

The scientific guarantor of this publication is Prof. Apostolos Karantanas.

### Statistics and biometry

One of the authors has significant statistical expertise.

### Study subjects or cohorts overlap

One of the two cohorts (UHH) used for the training of our deep learning models has been previously used to train another deep learning ensemble that distinguishes between avascular necrosis and transient osteoporosis. This work has no overlap with the present paper and has been published at https://doi.org/10.3390/diagnostics12081870. No staging of AVN had been attempted in the previous work and our current work does not attempt any diagnosis but only staging of the patients of the cohort. Therefore the work presented here is completely different without any overlap in aims or results.

### Methodology

- retrospective
- cross-sectional study
- multicenter study
