# Supplementary material for: Deep learning enables the differentiation between early and late stages of hip avascular necrosis
Source: Eur Radiol. 2023 Aug 15;34(2):1179–86. doi: 10.1007/s00330-023-10104-5 (PMC10853078; doi:10.1007/s00330-023-10104-5)

Supplementary Figure 1. Plots presenting the training/validation accuracy (A,C,E) and training/validation loss (B,D,F) for the finetuning of the final trainable layers of VGG-16 (A,B), Inception ResnetV2 (C,D), and Inception V3 (E,F).

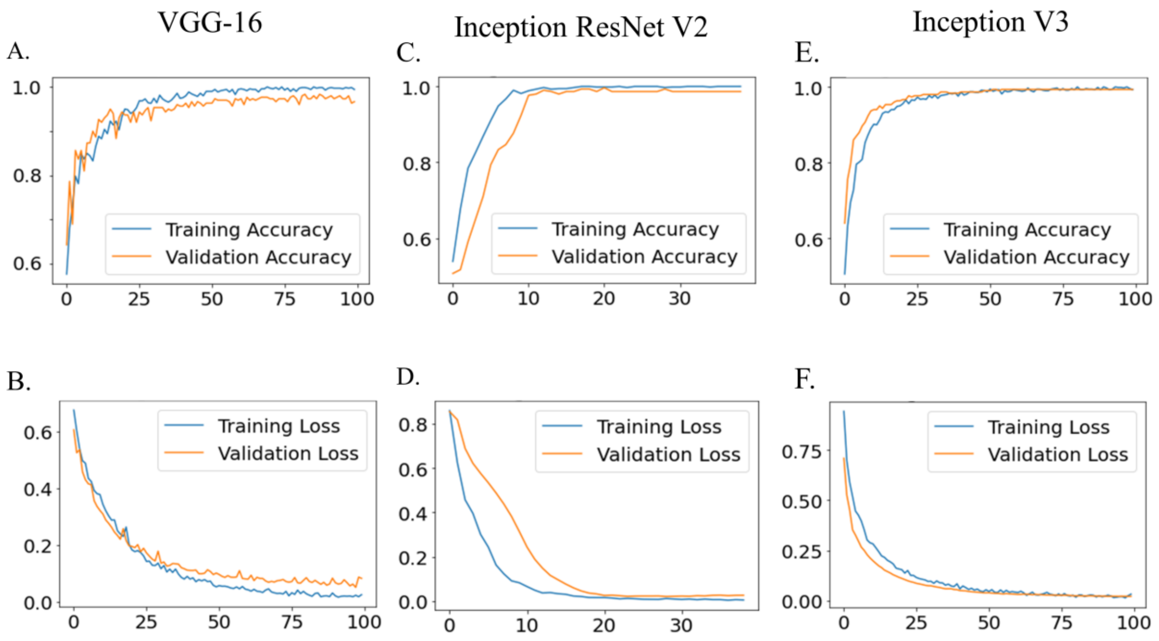

Supplement: Supplementary file 1 — Supplementary file1 (PDF 167 KB) [file 330_2023_10104_MOESM1_ESM.pdf]
